# Protective effect of wild polysaccharides extracted from *Ulva prolifera* on oxidative stress damage in valproic acid-induced neuronal cells

**DOI:** 10.3389/fphar.2026.1805470

**Published:** 2026-04-02

**Authors:** Xiaochun Xia, Qian Zhou, Hui Su, Li Liu, Jiaxuan Chen, Yiting Chen, Shaoqing Lin, Xulan Zhou, Juan Wang

**Affiliations:** 1 Department of Public Health and Medical Technology, Xiamen Medical College, Xiamen, China; 2 School of Public Health, Fujian Medical University, Fuzhou, China; 3 Department of Medical Record, Second Affiliated Hospital of Xiamen Medical College, Xiamen, China

**Keywords:** autism spectrum disorder (ASD), neuroprotective, oxidative stress, *Ulva prolifera* polysaccharides (PUPs), valproic acid (VPA)

## Abstract

**Background:**

Autism Spectrum Disorder (ASD) is a neurodevelopmental disorder, with prenatal exposure to valproic acid (VPA) being a recognized environmental risk factor for ASD, closely associated with its neurotoxic mechanism and oxidative stress. Marine green algae, such as *Ulva prolifera*, are rich sources of bioactive polysaccharides known for their antioxidant, anti-inflammatory, lipid-lowering, anti-tumor, and neuroprotective properties, with a core focus on antioxidant effects. Therefore, this study aims to investigate the protective effects and potential mechanisms of *Ulva prolifera* polysaccharides (PUPs) on oxidative stress damage induced by VPA in mouse hippocampal neuronal HT22 cells.

**Method:**

The primary structure of PUPs was determined through infrared spectroscopy, liquid chromatography, and gel permeation chromatography. PUPs intervention in the VPA-induced HT22 oxidative damage cell model was used to measure oxidative stress factor levels.

**Results:**

PUPs were characterized as composite polysaccharides with α-glycosidic bonds, sulfate ions, and aldonic acids, containing mannose, rhamnose, glucuronic acid, glucose, galactose, and xylose. The main molecular weight (Mw) was 2.124 kDa (80.535%). *In vitro* experiments revealed that PUPs significantly increased cell viability in the VPA model, enhanced intracellular SOD and CAT activities to boost antioxidant capacity, and concurrently reduced ROS levels and MDA content. Western blot analysis revealed that PUPs increased Nrf2, HO-1, and IκBα levels, while decreasing the expression levels of Keap1, p-NF-κB-p65/NF-κB-65, p-Erk/Erk, p-p38/p38, and p-JNK/JNK.

**Conclusions:**

PUPs demonstrate a significant protective effect against VPA-induced oxidative stress damage in HT22 neuronal cells. As a natural antioxidant, PUPs hold promising potential for the prevention and adjunct treatment of ASD-related neuronal oxidative stress damage.

## 1 Introduction

Autism Spectrum Disorder (ASD) is a neurodevelopmental disorder that typically emerges in infancy or early childhood. Its primary features include persistent deficits in social communication and interaction, as well as restricted, repetitive patterns of behavior, interests, or activities (Association, 2013). In recent years, there has been a significant increase in the global prevalence of ASD, imposing substantial economic and psychological burdens on families and society (Maenner et al., 2020). The Autism and Developmental Disabilities Monitoring (ADDM) Network of the Centers for Disease Control and Prevention (CDC) reveals an increase in the prevalence of ASD from 14.6‰ to 32.2‰ between 2012 and 2022 (Maenner et al., 2023). This may not only be attributed to broadening diagnostic criteria and increased public awareness, but also reflects the intricate interplay between genetic susceptibility and modern environmental exposure factors (Love et al., 2024). Although genetic factors play a predominant role in the development of ASD, mounting evidence suggests that environmental factors, particularly adverse exposures during pregnancy, are also significant risk factors.

Numerous epidemiological and clinical studies have consistently demonstrated that prenatal use of valproic acid (VPA) significantly increases the risk of congenital malformations, such as neural tube defects, and neurodevelopmental disorders, including ASD, in offspring (Ornoy, 2009). However, VPA is a widely used broad-spectrum antiepileptic drug and mood stabilizer (Michele et al., 2019). Prenatal VPA exposure has emerged as one of the most commonly utilized and reliable animal models for studying the pathophysiological mechanisms of ASD and screening potential intervention drugs (Nicolini and Fahnestock, 2018; Wang et al., 2025). The neurotoxic mechanism of VPA is complex, involving various aspects, including its role as a histone deacetylase (HDAC) inhibitor affecting epigenetic regulation of genes (Göttlicher et al., 2001), disrupting neurotransmitter balance (Rinaldi et al., 2007; Banerjee et al., 2013), inducing pathological changes in synaptic ultrastructure (Gąssowska-Dobrowolska et al., 2020), and causing dysbiosis of gut microbiota (Zhong et al., 2023). Induction of oxidative stress is considered a crucial step in neurodevelopmental damage caused by it. VPA is able to increase intracellular reactive oxygen species (ROS) production, deplete endogenous antioxidants, leading to lipid peroxidation and DNA damage, ultimately activating the apoptotic pathway, impairing neuronal survival and function (Saadat et al., 2023). Oxidative stress is a key factor in the pathophysiology of autism spectrum disorders, and it forms a feedback loop with other mechanisms (Heberling et al., 2013). Therefore, identifying natural bioactive compounds that can effectively counteract VPA-induced oxidative stress damage may offer new strategies for prevention and treatment of ASD.

The ocean serves as a vast repository of biological resources, with green algae harboring a variety of unique bioactive compounds. *Ulva prolifera* (formerly *Enteromorpha prolifera*) (PU) belongs to the phylum Chlorophyta, class Ulvophyceae, order Ulvales, and genus *Ulva*. It is a type of green algae that is widely distributed in intertidal zones worldwide. It proliferates rapidly in eutrophic waters, often leading to extensive “green tides” that negatively impact coastal ecosystems and tourism (Zhou et al., 2010).

However, from a perspective of resource utilization, fucus contains polysaccharides rich in sulfate groups, known as *Ulva prolifera* polysaccharides (PUPs). Numerous studies have confirmed the diverse biological activities of PUPs, including immunomodulation (Wei et al., 2014), anti-inflammatory (Guo et al., 2020), anti-tumor (Wang et al., 2014), lipid-lowering (Ren et al., 2018), and gut microbiota regulation (Ouyang et al., 2022). Its remarkable feature lies in its powerful antioxidant capacity. Evaluation of antioxidant activity *in vitro* is a common method for screening natural antioxidants. PUPs were evaluated using various models including superoxide anion radical scavenging, hydroxyl radical scavenging, and metal ion (e.g., Fe^2+^) chelation abilities. The results demonstrated that PUPs exhibited significant scavenging effects on these radicals and effectively chelated metal ions, with their activity typically showing dose-dependency (Li et al., 2013; Xu et al., 2015). Various animal models have further confirmed the antioxidant capacity of PUPs *in vivo*, demonstrating an increase in the activities of antioxidant enzymes such as superoxide dismutase (SOD), catalase (CAT), and glutathione peroxidase (GSH-Px), while reducing the levels of the lipid peroxidation product malondialdehyde (MDA) (Lin et al., 2015; Guo et al., 2021).

The HT22 cell line of mouse hippocampal neurons is an immortalized neuronal cell line (Fogo et al., 2024). The hippocampus is a crucial brain region for social interaction, learning, and memory, and is also one of the most vulnerable regions in ASD models (Bozdagi et al., 2010). HT22 is commonly used as an *in vitro* model for studying neuronal oxidative stress and cell death mechanisms. This cell line lacks functional ionotropic glutamate receptors, rendering it insensitive to glutamate excitotoxicity. However, it can respond to glutamate-induced oxidative damage through non-receptor pathways and exhibits high sensitivity to oxidative stress, making it an ideal tool for studying the neuroprotective effects of antioxidants (Behl et al., 1997). Therefore, we chose HT22 cells to establish a cellular model of ASD oxidative stress.

Given the significant role of VPA-induced oxidative stress in ASD pathology and the excellent antioxidant activity of PUPs, this study hypothesized that PUPs could protect HT22 neuronal cells from VPA-induced oxidative stress damage. To examine this further, we established a VPA-induced HT22 cell injury model and assessed the protective effects of PUPs, as well as their influence on the Nrf2/Keap1, MAPK, and NF-κB signaling pathways related to oxidative stress. The aim is to provide experimental evidence for PUPs as a potential functional food or drug for the prevention or treatment of neurodevelopmental disorders such as ASD.

## 2 Materials and methods

### 2.1 Characterization of PUPs

#### 2.1.1 Extraction of PUPs

The PU powder used in this study was made from wild seaweed harvested from the sea area of Fuqing, Fujian, China. A suspension of PE powder for aquaculture was prepared by mixing it with distilled water at a ratio of 1:40 (weight-to-volume). The mixture underwent heating in a water bath at 80 °C for 4 h. The cooled extract was centrifuged at 5000 *g* for 20 min, and the supernatant was concentrated to one-third of its volume using a rotary evaporator. The precipitate obtained by precipitation at 4 °C with 95% ethanol (1:4, v/v) is referred to as PUPs in the experiment.

#### 2.1.2 Detection of monosaccharide composition of PUPs

Prepare standard solutions of various monosaccharides including glucose (Solarbio, Beijing, China), galactose (Sigma, Missouri, United States), arabinose, fucose, rhamnose, mannose, ribose, xylose, glucuronic acid, and galacturonic acid (Aladdin, Shanghai, China). Begin by derivatizing them with 600 μL of 0.3 mol/L NaOH and 600 μL of 0.5 mol/L 1-Phenyl-3-methyl-5-pyrazolone (PMP, Sigma, Missouri, United States) in methanol at 70 °C for 30 min. After cooling to room temperature, adjust the pH to neutral and dilute to 2 mL with deionized water. Extract three times with 1 mL chloroform, collect the aqueous layer, filter, sonicate, and proceed to analysis. Prepare standard curves for each monosaccharide at different concentrations (10, 20, 40, 80, 160, 320, 640, 1280 μg/mL) for quantification purposes.

Weigh 2 mg of polysaccharide sample, add 3 mL of trifluoroacetic acid (2 mol/L), seal, and hydrolyze at 120 °C for 2 h. Subsequently, under reduced pressure at 45 °C, gradually add small amounts of methanol to remove residual trifluoroacetic acid. Proceed with derivatization as done for the monosaccharide standard solution. Inject 20 μL for analysis using an analytical liquid chromatograph (Shimadzu LC-20AR, Kyoto, Japan) with a UV detector (Shimadzu SPD-20A, Kyoto, Japan), employing a C18 column (4.6 mm × 250 mm, 5 μm, Uranus 5u, Guangzhou, China). The mobile phase consists of acetonitrile (A) and 0.02 mol/L ammonium acetate (B) with a gradient elution profile (0–5 min, 16% A; 5–17 min, 16%–19% A; 17–30 min, 19%–22% A; 30–35 min, 22%–16% A; 35–50 min,16% A). The flow rate is 1.0 mL/min, column temperature is 30 °C, and detection wavelength is set at 254 nm.

#### 2.1.3 Determination of PUPs molecular mass

Dissolve 5 mg of dextran with molecular weight gradients (P5, P10, P20, P50, P100, P200, P400, and P800, Showa Denko, Tokyo, Japan) separately in 1 mL of 0.05M NaCl. Molecular weight measurements were performed using high-performance liquid chromatography (Thermo U3000, Waltham, MA, United States) and a refractive index detector (RID-20A, Shimadzu Instruments, Kyoto, Japan). The separation column was a gel column (8 × 300 mm, BRT105-103-101, Borui Saccharide Biotechnology, Yangzhou, China) with a mobile phase of 0.05 M NaCl, a flow rate of 0.5 mL/min, and a column temperature of 40 °C.

Dissolve 5 mg of PUPs in 1 mL of mobile phase, sonicate for 10 min, centrifuge at 12000 rpm for 10 min, filter through a 0.22 μm aqueous microfiltration membrane, and proceed with the analysis as described above. Calculate the weight-average molecular weight (Mw) and number-average molecular weight (Mn) of PUPs based on the dextran calibration curve.

#### 2.1.4 Infrared spectral analysis of the PUPs

The functional groups in PUPs were identified by recording the Fourier transform infrared (FT-IR) spectrum. A 2 mg sample was mixed with KBr, pressed into a pellet, and analyzed using a Fourier Transform Infrared Spectrometer FT-IR650 (Tianjin Gangdong Technology, Tianjin, China) across the 4000−400 cm^−1^ range.

### 2.2 Evaluation of radical scavenging activity of PUPs using DPPH and ABTS assays

The method for measuring the DPPH free radical scavenging ability of PUPs was modified based on the literature (Mirzapour-Kouhdasht et al., 2022). A 0.02 mg/mL DPPH methanol solution (Macklin, Shanghai, China), was prepared in the dark and used immediately. PUPs and ascorbic acid solutions were prepared using distilled water at concentrations of 0, 0.05, 0.1, 0.2, 0.4, 0.6, 0.8, 1.0, 2, 4, 6, 8, and 10 mg/mL. The DPPH methanol solution was mixed in a 1:1 ratio with PUPs or ascorbic acid and incubated at room temperature in the dark until the reaction reached equilibrium. Blank wells (distilled water with DPPH methanol solution), positive control wells (ascorbic acid with DPPH methanol solution), sample test wells (PUPs solution with DPPH methanol solution), and sample blank wells (PUPs with methanol solution) were set up. The calculation for the activity of the DPPH free radical scavenging sample in the positive control wells was as follows: DPPH scavenging rate (%) = [(A_blank − A_positive control)/A_blank] × 100%; The calculation for the activity of the DPPH free radical scavenging sample in the PUPs wells was as follows: DPPH scavenging rate (%) = [[A_blank − (A_test − A_control)]/A_blank] × 100%.

The ABTS free radical scavenging capacity assay kit (Solaibao, BC4775, Beijing, China) was used to assess the ABTS free radical scavenging ability of PUPs. PUPs and ascorbic acid solutions were prepared using the extraction solution, with concentrations similar to the DPPH experiment, following the reagent ratios provided in the instructions. The samples were incubated at room temperature in the dark until the reaction reached equilibrium. The setup included a blank well (distilled water and ABTS solution), a positive control well (ascorbic acid and ABTS solution), a sample test well (PUPs solution and ABTS solution), and a sample blank well (PUPs and diluent). The calculation of the ABTS free radical scavenging activity for the positive control well was as follows: ABTS scavenging rate (%) = [(A_blank − A_positive control)/A blank] × 100%; The calculation for the ABTS free radical scavenging activity of PUPs was as follows: ABTS scavenging rate (%) = [[A_blank − (A_test − A_control)]/A blank] × 100%.

### 2.3 Cell models and drug interventions

#### 2.3.1 Cell culture

HT22 cells obtained from the Shanghai Cell Bank (Shanghai, China) were maintained in DMEM complete (Gibco, Jiangsu, China) medium supplemented with 10% fetal bovine serum (Zeta Life, France) and 1% penicillin-streptomycin (Gibco, NY, United States) at 37 °C and 5% CO_2_ in a humidified incubator (Thermo Forma 371, Thermo Fisher, MA, United States). Experiments were performed using cells in logarithmic growth phase with confluency exceeding 80%.

#### 2.3.2 CCK-8 assay

Use the CCK8 assay to evaluate the toxicity of VPA on HT22 cells and the effects of PUPs on the model. Cells were seeded at a rate of 5 × 10^3^ cells per well in a 96-well plate, and after 24 h, HT22 cells were treated with 1.5 mM VPA (Sigma, P4543, Saint Louis, TX, United States) for 24 h to establish the model group. Different concentrations of PUPs (0, 25, 50, 75 μg/mL) were co-administered with VPA, and the cells were further incubated for 24 h. Each group consisted of 6 replicate wells. After 24 h of incubation, 10% CCK8 solution (Apexbio, K1018, Houston, TX, United States) was added, and the absorbance was measured at 450 nm using a microplate reader (TECAN Infinite®200Pro, Groedig, Austria). The experiment was performed in triplicate for statistical analysis.

#### 2.3.3 The protective effect of PUPs on oxidative stress damage induced by VPA in HT22 cells

In HT22 cells, an oxidative damage model induced by VPA, the protective effects of PUPs at concentrations of 25, 50, and 75 μg/mL were assessed. Briefly, HT22 cells were co-cultured with 1.5 mM VPA and treated with PUPs at concentrations of 25, 50, and 75 μg/mL for 24 h Levels of catalase (CAT), malondialdehyde (MDA), and superoxide dismutase (SOD) were measured according to the kit instructions (Abbkine, CAT: KTB1040, MDA: KTB1050, SOD: KTB1030, Wuhan, China).

Utilizing a reactive oxygen species (ROS) detection kit based on DCFH-DA (Beyotime, S0033S, Shanghai, China), measure intracellular reactive oxygen species (ROS) levels according to the manufacturer’s instructions. DCFH-DA enters the cells and is hydrolyzed to DCFH, which remains trapped inside the cells since DCFH cannot exit the membrane. ROS oxidizes DCFH to form fluorescent DCF, and the fluorescence of DCF reflects the intracellular ROS levels. After a 24-h drug treatment, collect HT22 cells, wash them once with room temperature PBS, dilute 10 µM DCFH-DA probe in basal culture medium at a 1:8000 ratio, and add the diluted probe to each group for incubation at 37 °C in the dark for 20 min. Post-incubation, thoroughly wash the cells twice with cold PBS to remove excess dye. Analyze the fluorescence intensity of DCF using the FITC channel of a flow cytometer (NovoCyte 2050R, San Diego, CA, United States). The mean fluorescence intensity (MFI) is utilized to quantify ROS levels.

#### 2.3.4 Western blotting (WB)

Cells were manually homogenized using the RIPA buffer (Cell Signaling Technology, Danvers, MA, United States), in the presence of protease inhibitor (PMSF). Protein concentration was estimated using the BCA method. An equal amount of protein was separated on a 8%–12% SDS-PAGE gel. Transfer the sample proteins to polyvinylidene difluoride (PVDF) and seal with a blocking solution for 1 h. Subsequently, primary antibodies against NRF-2 (1:1000, Cell Signaling Technology, Danvers, MA, United States), Keap1 (1:1000, Cell Signaling Technology, Danvers, MA, United States), HO-1 (1:1000, Cell Signaling Technology, Danvers, MA, United States), IκBα (1:1000, Cell Signaling Technology, Danvers, MA, United States), NF-κB-p65 (1:1000, UpingBio, Hangzhou, China), p-NF-κB-p65 (1:1000, UpingBio, Hangzhou, China), Erk (1:1000, Cell Signaling Technology, Danvers, MA, United States), p-Erk (1:1000, Cell Signaling Technology, Danvers, MA, United States), p-p38 (1:1000, Cell Signaling Technology, Danvers, MA, United States), p38 (1:1000, Cell Signaling Technology, Danvers, MA, United States), JNK (1:1000, Cell Signaling Technology, Danvers, MA, United States), p-JNK (1:1000, Cell Signaling Technology, Danvers, MA, United States), or GAPDH (1:10,000, Proteintech, Rosemont, IL, United States) were incubated at 4 °C overnight. Then, incubate with goat anti-rabbit or anti-mouse immunoglobulin G secondary antibody conjugated with horseradish peroxidase (1:10,000, Pierce, New York, United States) at room temperature for 1 h. After exposure to ECL reagent, the bands were visualized and imaged using an ECL imaging system (Bio-Rad, Berkeley, California, United States).

### 2.4 Statistical analyses

The outcomes were indicated as mean ± SD. The study conducted tests for normality (Shapiro-Wilk test or D'Agostino and Pearson test) and homogeneity of variances (Brown-Forsythe test). For groups meeting the assumptions, perform one-way analysis of variance, followed by Dunnett’s multiple comparison tests with SPSS Statistics 27 software (IBM Corporation, Armonk, New York, United States). Due to a set of data not meeting normal distribution, utilize a nonparametric test (Kruskal-Wallis H test) and conduct Dunn’s multiple comparison tests on the p-JNK/JNK variable. Statistical significance was established at *P* < 0.05. The * symbols represent comparisons with the model groups. Drawing and statistical analysis were conducted using GraphPad Prism 9.0.5 and OriginPro 2024.

## 3 Results

### 3.1 Primary structural of PUPs

#### 3.1.1 Monosaccharide composition of PUPs

**FIGURE 1:**
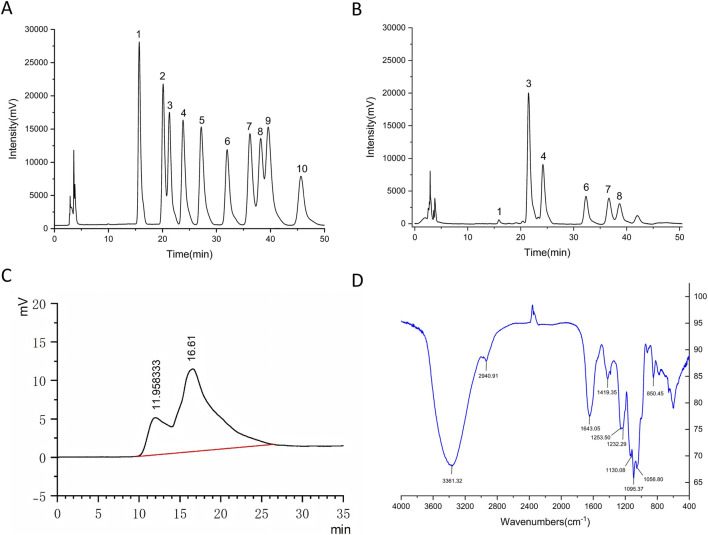
Structural characterization of PUPs. **(A)** HPLC chromatogram of a mixed monosaccharide standard, showing the retention times of 10 reference monosaccharides: 1. mannose; 2. ribose; 3. rhamnose; 4. glucuronic acid; 5. galacturonic acid; 6. glucose; 7. galactose; 8. xylose; 9. arabinose; 10. fucose. **(B)** Monosaccharide composition analysis of PUPs by HPLC, identifying the presence of seven monosaccharides: 1. mannose; 2. rhamnose; 4. glucuronic acid; 6. glucose; 7. galactose; 8. xylose. **(C)** Molecular weight distribution of PUPs determined by High Performance Gel Permeation Chromatography (HPGPC). **(D)** FTIR spectra of PUPs, showing characteristic functional groups and glycosidic bond vibrations in the range of 4000-400 cm^−1^, indicating typical polysaccharide structural features.

Based on the chromatographic data of monosaccharide standards, the standard curve equations for 10 monosaccharide standards were calculated using mass concentration x (mg/mL) and peak area y as the x and y coordinates, respectively. The results indicate that the standard curve data is excellent, with correlation coefficients R^2^ close to 99% (Table 1). By comparing the chromatograms of mixed monosaccharide standards and PUPs (Figures 1A,B), it was found that the monosaccharide composition of PUPs mainly consists of mannose, rhamnose, glucuronic acid, glucose, galactose and xylose in proportions of 0.013: 0.833: 0.479: 0.249: 0.240: 0.230.

**TABLE 1:** Standard curve and regression coefficient of monosaccharide standards.

Monosaccharide	Retention time	Regression equation	R^2^
Mannose	15.688	Y = 8602 × X − 97388	0.9996
Ribose	20.111	Y = 8040 × X − 6577	0.9998
Rhamnose	21.268	Y = 5736 × X − 96663	0.9989
Glucuronic acid	23.802	Y = 7567 × X − 50538	0.9997
Galacturonic acid	27.175	Y = 7911 × X − 23976	0.9997
Glucose	31.966	Y = 6121 × X − 106648	0.9990
Galactose	36.196	Y = 9488 × X − 110033	0.9997
Xylose	38.189	Y = 6706 × X − 90836	0.9997
Arabinose	39.568	Y = 8725 × X − 130522	0.9994
Fucose	45.624	Y = 5686 × X + 9175	0.9990

#### 3.1.2 Molecular weight of PUPs

Analysis using High-Performance Gel Permeation Chromatography (HPGPC) revealed a bimodal molecular weight distribution in the PUPs. The main peak (retention time 16.612 min) had a number-average molecular weight (Mn) of 2.618 kDa, weight-average molecular weight (Mw) of 2.124 kDa, and a polydispersity index (Mw/Mn) of 0.81, indicating a narrow distribution with a relative peak area of 80.535%, representing the major component of the sample (Figures 1C). The minor peak (retention time 11.958 min) had Mn of 1563.338 kDa, Mw of 1309.722 kDa, and Mw/Mn of 0.84. Despite its significantly higher molecular weight compared to the main peak, it only accounted for 19.465% of the relative peak area, suggesting the presence of a small amount of high molecular weight components in the sample. Overall, the polysaccharide was predominantly composed of low molecular weight components (2 kDa), with a broad molecular weight distribution.

#### 3.1.3 Analysis of PUPs using infrared spectroscopy

FT-IR spectroscopy was used to analyze the functional groups and vibration modes in polysaccharides (Figure 1D). A broad peak was observed in the absorption band at 3600–3200 cm^-1^, characteristic of sugar substances. Specifically, the peak at 3361 cm^-1^ corresponds to the stretching vibration of O-H, indicating the presence of numerous hydroxyl functional groups in the sample. The peak at 2940 cm^-1^ corresponds to the stretching vibration of C-H, further confirming the presence of a sugar ring structure. An absorption peak at 1643 cm^-1^ may be attributed to the stretching vibration of C=O, suggesting the presence of a small amount of oxygen-containing functional groups such as carboxyl or acetyl, indicating the presence of aldonic acids in the sample. Peaks at 1419 cm^-1^ and 1130 cm^-1^ may be attributed to the stretching vibration of C-O, reflecting the typical features of glycosidic bonds in polysaccharide molecules. The peak at 1095 cm^-1^ may be attributed to the symmetric stretching vibration of sulfate ions. Peaks at 1253 cm^-1^, 1232 cm^-1^, and 1056 cm^-1^ may be attributed to the bending vibration of O-H, related to the hydroxyl conformation in the sugar ring. An absorption peak at 850 cm^-1^ may be attributed to the bending vibration of α-anomeric C-H. These findings indicate that PUPs are complex polysaccharides containing hydroxyl groups, aldonic acids, sulfate ions and α-glycosidic bonds.

### 3.2 Effect of PUPs on the DPPH and ABTS radical scavenging rates

**FIGURE 2:**
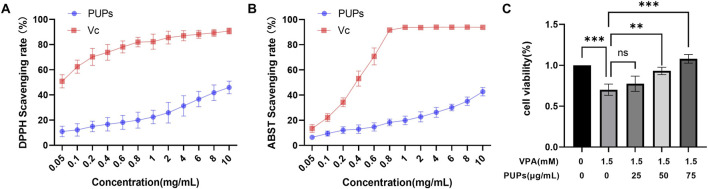
Evaluation of PUPs’ *in vitro* antioxidant capacity and their protective effect on VPA-induced HT22 cell viability. **(A)** Effect of PUPs at concentrations ranging from 0.05 to 10 mg/mL on the radical scavenging rates of DPPH. **(B)** Effect of PUPs at concentrations ranging from 0.05 to 10 mg/mL on the radical scavenging rates of ABTS. **(C)** Effects of different concentrations of PUPs on the viability of HT22 cells treated with 1.5 µM VPA compared with the model group (VPA-treatment group). Data are presented as mean ± SD. **P < 0.01, ***P < 0.001 vs. the model group (VPA treatment group).

The 2,2-diphenyl-1-picrylhydrazyl (DPPH) is a relatively stable nitrogen-centered free radical with strong absorption at 517 nm. Therefore, changes in absorbance at this wavelength can indicate the sample’s free radical scavenging ability. As shown in Figure 2A, PUPs exhibited significant free radical scavenging activity in the concentration range of 0.05–10.0 mg/mL, with DPPH scavenging increasing from 10.98% ± 4.15% to 45.94% ± 4.98%. Similar results were observed in the ABTS free radical scavenging assay. The 2,2′-azino-bis-3-ethylbenzothiazoline-6-sulfonic acid (ABTS) forms stable blue-green ABTS radicals upon oxidation, with a maximum absorption peak at 405 nm, which can react with antioxidant components causing the system to fade. As depicted in Figure 2B, as the concentration of PUPs increased from 0.05 to 10.0 mg/mL, the ABTS scavenging rate gradually rose from 6.49% ± 1.69% to 42.68% ± 3.36%.

### 3.3 Effect of PUPs on cell viability and antioxidant activity in VPA-Induced HT22 cells

#### 3.3.1 Effects of PUPs on cell viability in VPA-Induced HT22 cells

In preliminary experiments, it was found that 0.5 mM VPA significantly induces oxidative stress in HT-22 cells and reduces their viability. Therefore, we utilized this concentration to establish a subsequent oxidative stress model in HT-22 cells. Based on CCK8 experiments, we observed that different concentrations of PUPs increased the cell viability of HT-22 cells. Specifically, PUPs at concentrations of 50–75 μg/mL significantly improved the viability of VPA-induced HK-22 cells (Figure 2C).

#### 3.3.2 Effect of PUPs on the levels of catalase, malondialdehyde, superoxide dismutase, and reactive oxygen species in VPA-Induced HT22 cells

**FIGURE 3:**
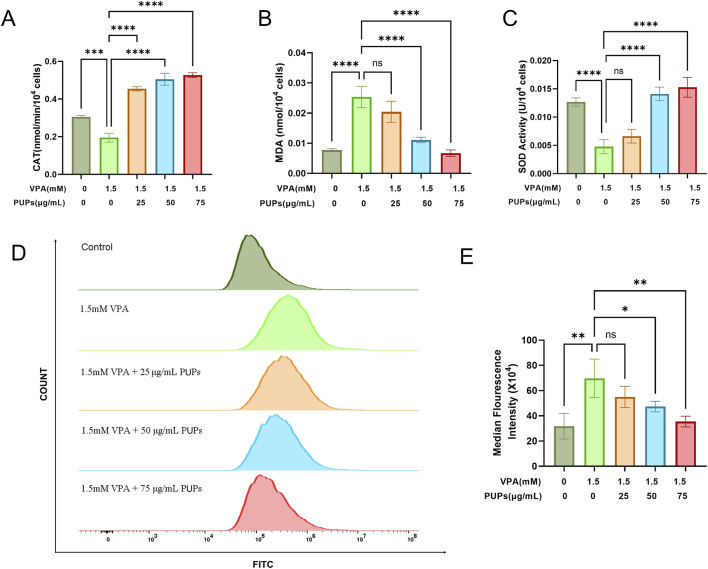
PUPs modulates oxidative stress in VPA induced HT22 Cells. **(A)** Effects of PUPs on CAT, **(B)** MDA, and **(C)** SOD levels in VPA-induced HT22 cells. **(D)** Flow cytometry to detect the effect of PUPs on ROS levels. **(E)** Histogram of the relative mean fluorescence intensity of ROS in each group. Data are presented as mean ± SD. *P < 0.05, **P < 0.01, ***P < 0.001, ****P < 0.0001 vs. the model group (VPA treatment group).

As shown in Figures 3A–C, VPA induction in HT22 cells significantly increased MDA levels, while PUPs dose-dependently reduced MDA content, with the highest dose group showing the most pronounced effect. Conversely, VPA induction significantly decreased antioxidant substances like CAT and SOD, whereas PUPs treatment significantly elevated their levels. Additionally, as depicted in Figures 3D,E, flow cytometry analysis revealed that VPA-induced ROS fluorescence in HT22 cells increased compared to the control group, while PUPs treatment enhanced intracellular ROS clearance capacity, leading to a gradual decrease in ROS fluorescence in a dose-dependent manner, indicating that PUPs enhanced the cell’s ability to eliminate ROS with increasing doses. Find the original flow cytometry image in Supplementary Figure S1. Effect of PUPs on the classic pathway Keap1/Nrf2/HO-1 of oxidative stress in VPA-induced HT22 cells.

#### 3.3.3 Effect of PUPs on the classic pathway Keap1/Nrf2/HO-1 of oxidative stress in VPA-induced HT22 cells

**FIGURE 4:**
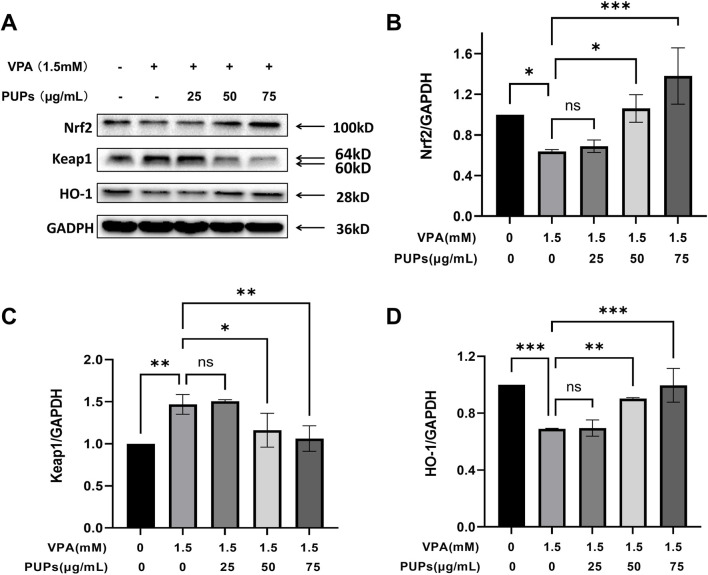
Western blot analysis revealed the effects of different concentrations of PUPs on the classical oxidative stress Keap1/Nrf2/HO-1 pathway in HT22 cells (*n* = 3/each group). **(A)** The Western blot image of Keap1/Nrf2/HO-1 proteins. **(B)** Protein expression levels of Nrf2. **(C)** Protein expression levels of Keap1. **(D)** Protein expression levels of HO-1. Data are presented as mean ± SD. *P < 0.05, **P < 0.01, ***P < 0.001 vs. the model group (VPA treatment group).

The Keap1-Nrf2-HO-1 signaling pathway is a crucial defense mechanism in cells to combat oxidative stress and maintain redox homeostasis. By regulating the expression of antioxidant enzymes, it enhances the cell’s ability to eliminate free radicals, thus protecting the cell from damage. As shown in Figures 4A–D; Supplementary Figure S2, the experimental findings show that intervention with VPA leads to a significant decrease in Nrf2 and HO-1 expression and a notable increase in Keap1 expression in HT22 cells. Treatment with PUPs in the VPA model cells results in a significant increase in Nrf2 and HO-1 expression and a marked decrease in Keap1 expression in HT22 cells at doses of 50–75 μg/mL, showing a certain level of dose dependency.

#### 3.3.4 Effect of PUPs on the NF-κB signaling pathways in VPA-induced HT22 cells

**FIGURE 5:**
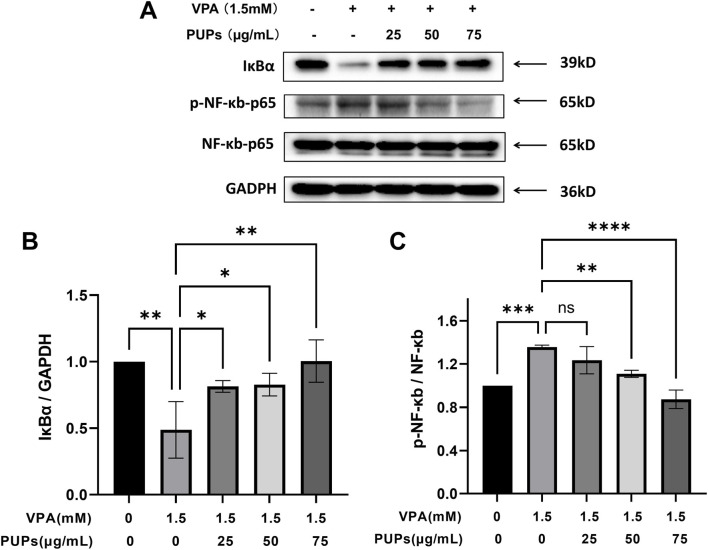
Western blot analysis revealed the effects of different concentrations of PUPs on the NF-κB signaling pathways in HT22 cells (*n* = 3/each group). **(A)** The Western blot image of IκBα, p-NF-κB, and NF-κB proteins. **(B)** Protein expression levels of IκBα. **(C)** Protein expression levels of p-NF-κB/NF-κB. Data are presented as mean ± SD. *P < 0.05, **P < 0.01, ***P < 0.001, ****P < 0.0001 vs. the model group (VPA treatment group).

Due to its role in regulating inflammation, immune response, and cell survival, the IκBα-NF-κB pathway is a crucial signaling pathway. Oxidative stress can directly activate this pathway, leading to a harmful cycle of “oxidative stress-inflammation.” Therefore, this study further examined the IκBα-NF-κB pathway. As shown in Figures 5A–C; Supplementary Figure S3, it was observed that VPA significantly reduced the expression of IκBα while increasing the expression of p-NF-κB/NF-κB. Intervention with 25–75 μg/mL of PUPs consistently led to a significant increase in IκBα expression. Moreover, treatment with 50–75 μg/mL of PUPs significantly decreased the expression of p-NF-κB/NF-κB.

#### 3.3.5 Effect of PUPs on the MAPK signaling pathways in VPA-induced HT22 cells

**FIGURE 6:**
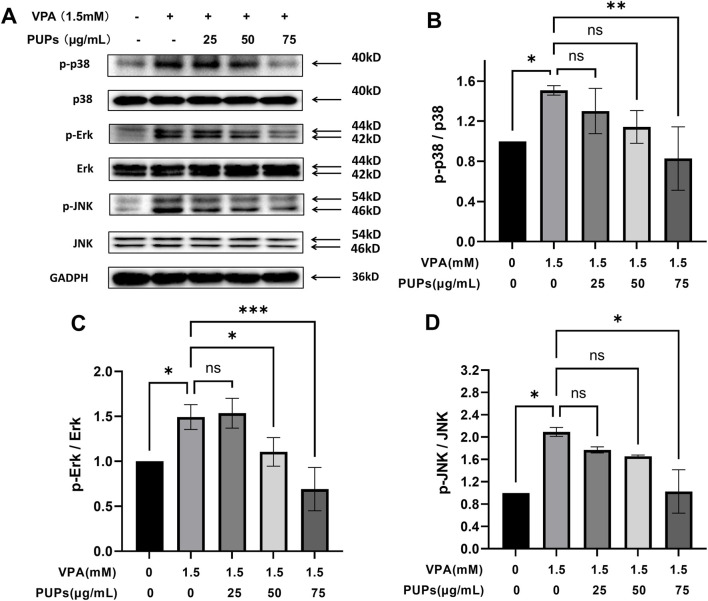
Western blot analysis revealed the effects of different concentrations of PUPs on the MAPK signaling pathways in HT22 cells (*n* = 3/each group). **(A)** The Western blot image of p-p38, p38, p-Erk, Erk, p-JNK, and JNK proteins. **(B)** Protein expression levels of p38/p38. **(C)** Protein expression levels of p-Erk/Erk. **(D)** Protein expression levels of p-JNK/JNK. Data are presented as mean ± SD. *P < 0.05, **P < 0.01, ***P < 0.001, ****P < 0.0001 vs. the model group (VPA treatment group).

One of the core mechanisms for cells to sense and respond to oxidative stress is the MAPK signaling pathway. It not only rapidly transmits stress signals but also regulates cell fate (such as survival, apoptosis, inflammation), playing a crucial role in the development of various diseases. Research on the MAPK family mainly focuses on detecting the three sub-pathways: ERK, JNK, and p38. As shown in Figures 6A–D; Supplementary Figure S4, it has been observed that VPA significantly increases the expression of p-p38/p38, p-Erk/Erk, and p-JNK/JNK. With PUPs intervention, the expression of p-p38/p38, p-Erk/Erk, and p-JNK/JNK is downregulated and shows a certain level of dose dependency.

## 4 Discussion

ASD is a complex neurodevelopmental condition with a multifactorial etiology involving genetic and environmental factors (Campos et al., 2025). The association between the use of VPA as a widely used antiepileptic drug during pregnancy and an increased risk of neural tube defects and ASD has been well established (Liang et al., 2024), with its induced neurotoxicity closely related to oxidative stress (Tung and Winn, 2011; Asghar et al., 2025). Several studies have shown that one of the neurotoxic mechanisms of valproic acid (VPA) is through disrupting mitochondrial function, leading to excessive generation of reactive oxygen species (ROS) and depletion of intracellular antioxidant defense substances, such as glutathione (GSH), catalase (CAT), and superoxide dismutase (SOD) (Heidari et al., 2018; Hansen et al., 2021; Asghar et al., 2025). This oxidative/antioxidant imbalance can lead to lipid peroxidation, protein damage, and DNA breakage, ultimately resulting in neuronal dysfunction and cell apoptosis, which is believed to be the pathological basis of VPA-related neurodevelopmental disorders (including ASD) (Wang et al., 2024; Zhou et al., 2025).

In this study, we observed a significant decrease in HT22 cell viability upon VPA treatment, accompanied by a sharp increase in intracellular ROS and lipid peroxidation product MDA levels, as well as a decrease in key antioxidant enzyme SOD and CAT activities. These results are consistent with previous findings (Chaudhary and Parvez, 2012; Aranarochana et al., 2021), establishing a reliable *in vitro* model of VPA-induced neuronal oxidative stress damage for evaluating the protective effects of PUPs in subsequent studies.

PU, a common edible green algae, has been shown to possess various biological activities, particularly strong antioxidant capabilities (Wassie et al., 2021). Previous studies have shown that the antioxidant activity of polysaccharides is closely related to their molecular weight, monosaccharide composition, glycosidic bond type, and sulfate content (Meng et al., 2015). Structural analysis reveals that the PUPs utilized in this study are complex polysaccharides containing sialic acid and α-glycosidic bonds, with the predominant component being a low molecular weight polysaccharide of 2.124 kDa. The molecular weight of polysaccharides is a key factor influencing their biological activity. Generally, polysaccharides with lower molecular weight have better water solubility and higher exposure of active groups, thereby exhibiting stronger antioxidant activity. Typically, polysaccharides with lower molecular weight have better water solubility and bioavailability, making it easier for them to enter cells and exert their effects (Liu et al., 2010), which may explain the excellent protective effect shown by PUPs in this study. The antioxidant effect of PUPs may also be attributed to their sulfuric acid groups and aldonic acid groups, which can effectively capture free radicals and neutralize oxidative stress, thereby protecting cells from damage and promoting the activity of antioxidant enzymes in the body (Sun et al., 2015).

Several studies have demonstrated that PUPs can effectively scavenge various free radicals and enhance the body’s antioxidant defense system (Li et al., 2013; Xu et al., 2015). This study found that pre-treatment with PUPs significantly reversed the decrease in cell viability induced by VPA, while also significantly reducing ROS and MDA levels in the cells. Importantly, PUUPs restored the activity of SOD and CAT that were inhibited by VPA. This suggests that the neuroprotective effect of PUPs is at least partially achieved through its direct antioxidant activity and enhancement of endogenous antioxidant defense capabilities.

To further investigate the antioxidant mechanism of PUPs, we examined the changes in the Nrf2/Keap1 signaling pathway. Nrf2 is a core transcription factor that helps cells resist oxidative stress and inflammation. Under normal physiological conditions, it binds to the inhibitory protein Keap1 and is degraded. When cells are exposed to oxidative stress, Nrf2 dissociates from Keap1, enters the nucleus, and activates the expression of downstream antioxidant genes such as HO-1 (Asghar et al., 2025). The Nrf2 pathway plays a crucial role in VPA-induced neurotoxicity models, and its activation is considered an effective protective strategy (Zhao et al., 2022). This study found that PUPs treatment significantly reduced the protein levels of Keap1, while upregulating the expression of Nrf2 and its downstream target protein HO-1. This suggests that PUPs may enhance cellular antioxidant capacity by promoting the dissociation of Nrf2 from Keap1 and activating the Nrf2 signaling pathway. This finding is similar to the mechanisms of action of other natural antioxidants in VPA models, providing strong molecular evidence for the neuroprotective effects of PUPs (Abbasalipour et al., 2022).

Reactive oxygen species (ROS), as byproducts of cellular metabolism and important secondary messengers, have been confirmed to be key regulatory molecules upstream of the NF-κB signaling pathway (Asghar et al., 2025). NF-κB plays a central role in various physiological and pathological processes such as inflammation, immune regulation, cell proliferation, and apoptosis. In the resting state, the inactive trimeric complex is formed by the binding of IκBα to NF-κB, retaining it in the cytoplasm (DiDonato et al., 1995). When cells are exposed to external stimuli, such as inflammation or oxidative stress, IκBα undergoes phosphorylation and degradation, leading to the activation of the upstream IκB kinase (IKK) complex (Li et al., 2024). The activated IKK catalyzes the phosphorylation of two crucial serine residues (Ser32 and Ser36) at the N-terminus of IκBα protein (Cohen et al., 1998). The phosphorylated IκBα is then recognized by E3 ubiquitin ligase, ubiquitinated, and ultimately degraded by the 26S proteasome (Moynagh, 2005). The activation of NF-κB by many activators can induce the production of ROS (Nakajima and Kitamura, 2013), whereas antioxidants can effectively inhibit this activation (Shi et al., 2000; Han et al., 2001; Xu et al., 2024). External administration of H2O2 can directly activate NF-κB (Takada et al., 2003). Pre-treating cells with antioxidants such as N-acetylcysteine (NAC) can significantly inhibit NF-κB activation induced by various stimuli like TNF-α and LPS (Jiang et al., 2018; Dos-Santos-Pereira et al., 2020). This research has shown that PUPs can upregulate the expression of IκBα, significantly inhibit the phosphorylation of NF-κB p65 subunit, thereby blocking the activation of NF-κB. Studies indicate that PUPs play a role in oxidative stress and inflammatory responses, with a close association between oxidative stress and inflammatory reactions. Both mechanisms mutually promote each other, leading to neuronal damage.

Similarly, the phosphorylation activation of the MAPK family (including Erk, p38, and JNK) also plays a crucial role in cell apoptosis and inflammation. In most cases, ROS acts as a potent activator of Erk, p38, and JNK. Various external stimuli, such as UVB radiation (Teng et al., 2025), cadmium heavy metal (Cao et al., 2021), herbicidesc (Lin et al., 2025), and nanomaterials (Li et al., 2012), can induce an increase in intracellular ROS levels, leading to the phosphorylation of Erk, p38, and JNK, ultimately triggering cell apoptosis, inflammatory responses, or ferroptosis as cellular stress outcomes. Upstream of MAPK activation, ROS plays a crucial role. Various antioxidants can effectively inhibit the excessive activation of the MAPK pathway by scavenging ROS or enhancing cellular antioxidant capacity. N-acetylcysteine (NAC), as a precursor of glutathione and a direct ROS scavenger, exerts protective effects by inhibiting the phosphorylation of ERK, JNK, and p38 (El-Najjar et al., 2010; Zhang et al., 2011). In this study, PUPs significantly inhibit the phosphorylation levels of three key proteins (Erk, p38, JNK) in the MAPK family induced by VPA, which may primarily stem from the antioxidant properties of PUPs.

Due to its role as an upstream signaling molecule activating the MAPK and NF-κB pathways, PUPs likely exert their inhibitory effects on these pathways as a direct result of clearing ROS and alleviating oxidative stress. By suppressing these pro-inflammatory and pro-apoptotic signaling pathways, PUPs can more comprehensively protect neurons from the toxic damage induced by VPA. Furthermore, the regulatory ability of PUPs on these pathways has been confirmed in other studies as well (Wei et al., 2014; Feng et al., 2023). Hence, the neuroprotective effect of PUPs is a synergistic outcome of their antioxidant and anti-inflammatory activities, achieved through a multi-pathway, multi-target approach.

**FIGURE 7:**
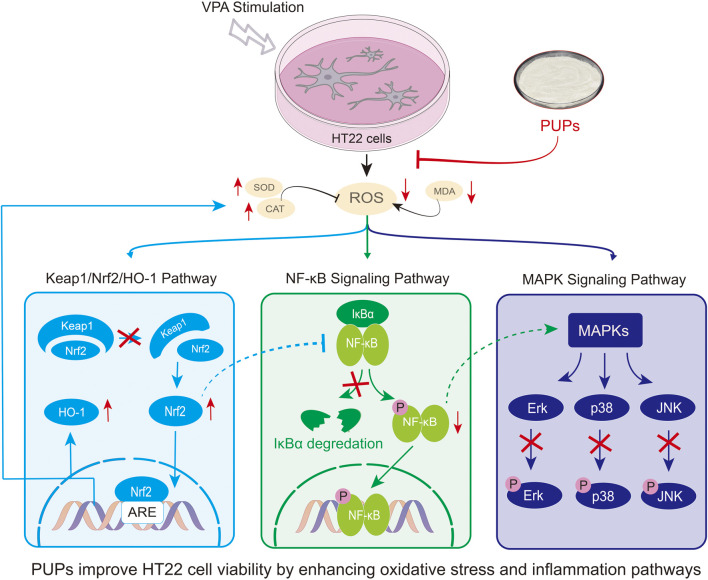
Mechanism diagram of VPA-induced HT22 oxidative stress model protected by PUPs.

Overall, this study reveals for the first time the protective role of PUPs in the VPA-induced neuronal oxidative stress model and elucidates that its mechanism of action involves key signaling pathways such as Nrf2, NF-κB, and MAPK (Figure 7). Considering that seaweed is a safe, widely available natural edible resource, PUPs, as a natural antioxidant, show great potential in preventing neurodevelopmental disorders related to prenatal oxidative stress, such as ASD. Furthermore, PUPs have been reported to possess various biological activities such as immune regulation (Kim et al., 2011) and anti-inflammatory properties (Feng et al., 2023), which may synergistically enhance their neuroprotective effects. This not only provides new experimental evidence for the application of PUPs as neuroprotective agents but also offers new insights into the prevention and treatment of neurodevelopmental disorders related to oxidative stress such as ASD. However, this study has some limitations. Firstly, it was conducted only on *in vitro* cell models, and further validation is required to determine if the results can be fully translated into the complex physiological environment *in vivo*. Secondly, although we characterized the molecular weight and basic structure of PUPs, the exact structural elements or active components responsible for their biological activities remain unclear. Lastly, while the VPA model is widely used, the HT22 immortalized cell line, with its physiological characteristics differing from primary neurons or the complex neural environment *in vivo*.

Based on the findings and limitations of this study, future research directions could include the following aspects. Firstly, it is important to validate the neuroprotective effects of PUPs in primary neurons cultured with VPA induction, brain organoids, or VPA-induced animal models of autism, and assess their potential for improving core symptoms such as social impairments and repetitive behaviors. Secondly, further refinement in the isolation, purification, and structural identification of PUPs is needed to clarify their structure-activity relationship, laying the groundwork for developing more potent derivatives or analogs. Thirdly, in addition to antioxidant and anti-inflammatory properties, exploring whether PUPs exert their effects through other mechanisms, such as modulating the gut microbiota-brain axis, which is considered an emerging area in ASD pathophysiology, is warranted. Furthermore, investigating the synergistic effects of PUPs with other neuroprotective natural compounds, such as melatonin (Liang et al., 2024) or resveratrol (Santos-Terra et al., 2022), may also offer new strategies for intervening in ASD.

## 5 Conclusion

Research has shown that PUPs effectively protect HT22 neurons from oxidative stress induced by VPA. The mechanism involves enhancing endogenous antioxidant enzyme activity, scavenging reactive oxygen species (ROS), inhibiting the classical oxidative stress Keap1/Nrf2/HO-1 pathway, and suppressing the excessive activation of NF-κB and MAPK signaling pathways triggered by oxidative stress. These findings not only deepen the understanding of the neuroprotective mechanism of PUPs but also provide important scientific evidence for their use as a natural antioxidant in preventing and assisting in the treatment of oxidative stress-related neurodevelopmental disorders such as ASD.

## Data Availability

The original contributions presented in the study are included in the article/Supplementary Material, further inquiries can be directed to the corresponding authors.
