# Supplementary material for: Protective effect of wild polysaccharides extracted from Ulva prolifera on oxidative stress damage in valproic acid-induced neuronal cells
Source: Front Pharmacol. 2026 Apr 2;17:1805470. doi: 10.3389/fphar.2026.1805470 (PMC13083006; doi:10.3389/fphar.2026.1805470)
Supplement: Supplementary file 1 [file Supplementaryfile1.docx]

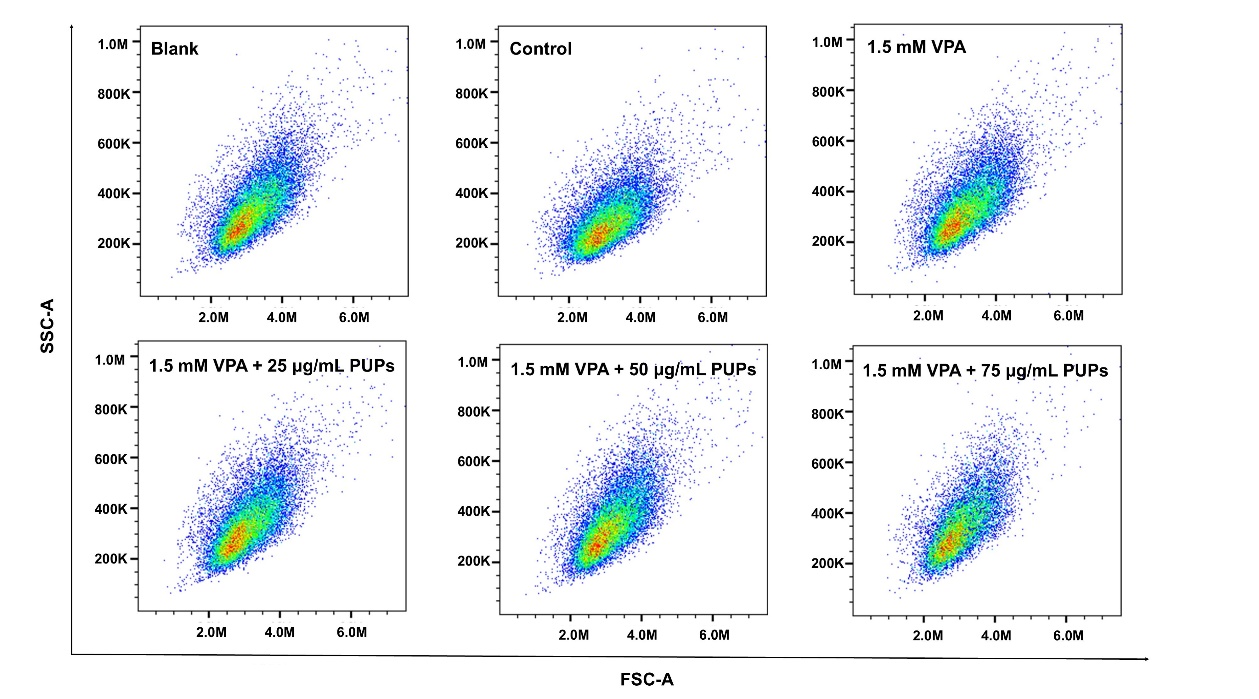
**Supplementary Fig1.** Original spectrum of reactive oxygen species (ROS) detected by flow cytometry.


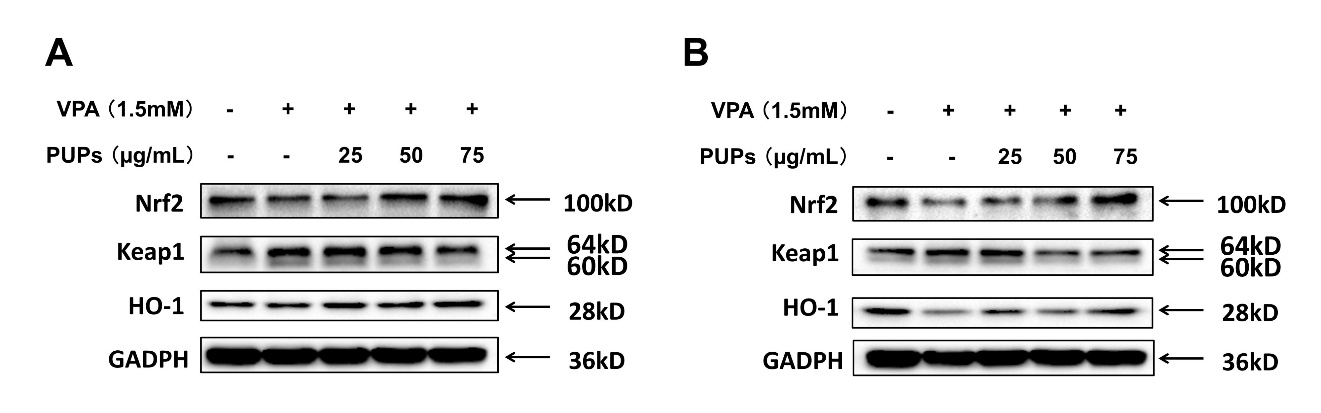


**Supplementary Fig2:** Western blot analysis demonstrated the effects of different concentrations of PUPs on the classical oxidative stress Keap1/Nrf2/HO-1 pathway in HT22 cells, which were also utilized for statistical analysis with two additional datasets.


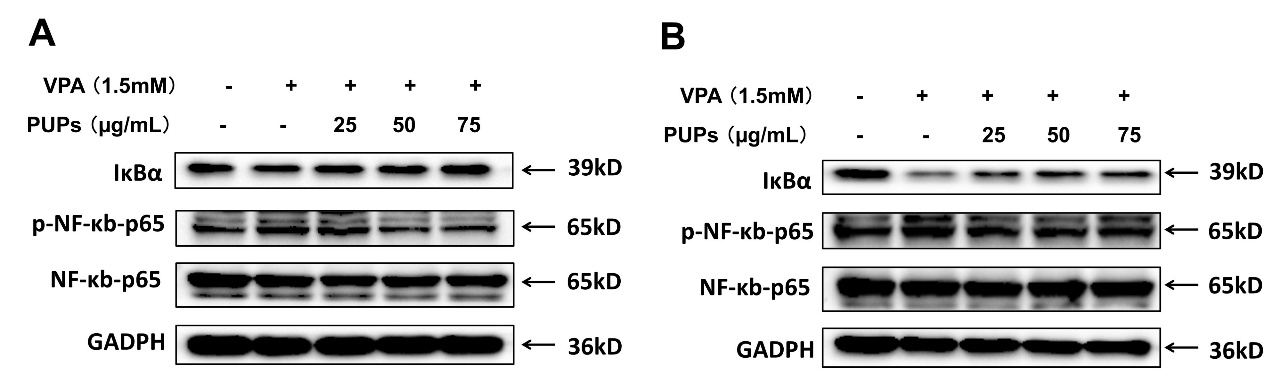


**Supplementary Fig3:** Western blot analysis revealed the effects of different concentrations of PUPs on the NF-κB signaling pathways in HT22 cells, which were also utilized for statistical analysis with two additional datasets.


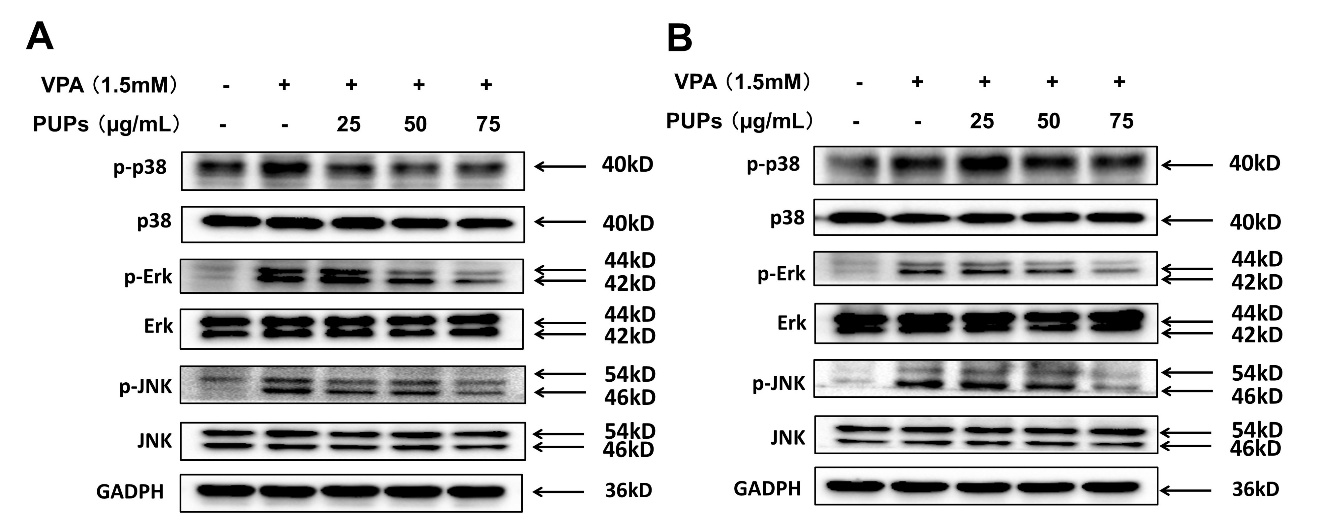


**Supplementary Fig4:** Western blot analysis revealed the effects of different concentrations of PUPs on the MAPK signaling pathways in HT22 cells, which were also utilized for statistical analysis with two additional datasets.
